# Supplementary material for: A Computational Workflow for Structure-Guided Design of Potent and Selective Kinase Peptide Substrates
Source: bioRxiv. 2025 Jul 5:2025.07.04.663216. Preprint. [Version 1] doi: 10.1101/2025.07.04.663216 (PMC12236476; doi:10.1101/2025.07.04.663216)
Supplement: 1 [file NIHPP2025.07.04.663216v1-supplement-1.pdf]

bioRxiv preprint doi: https://doi.org/10.1101/2025.07.04.663216; this version posted July 5, 2025. The copyright holder for this preprint (which was not certified by peer review) is the author/funder, who has granted bioRxiv a license to display the preprint in perpetuity. It is made available under aCC-BY 4.0 International license.

Supplementary Figures

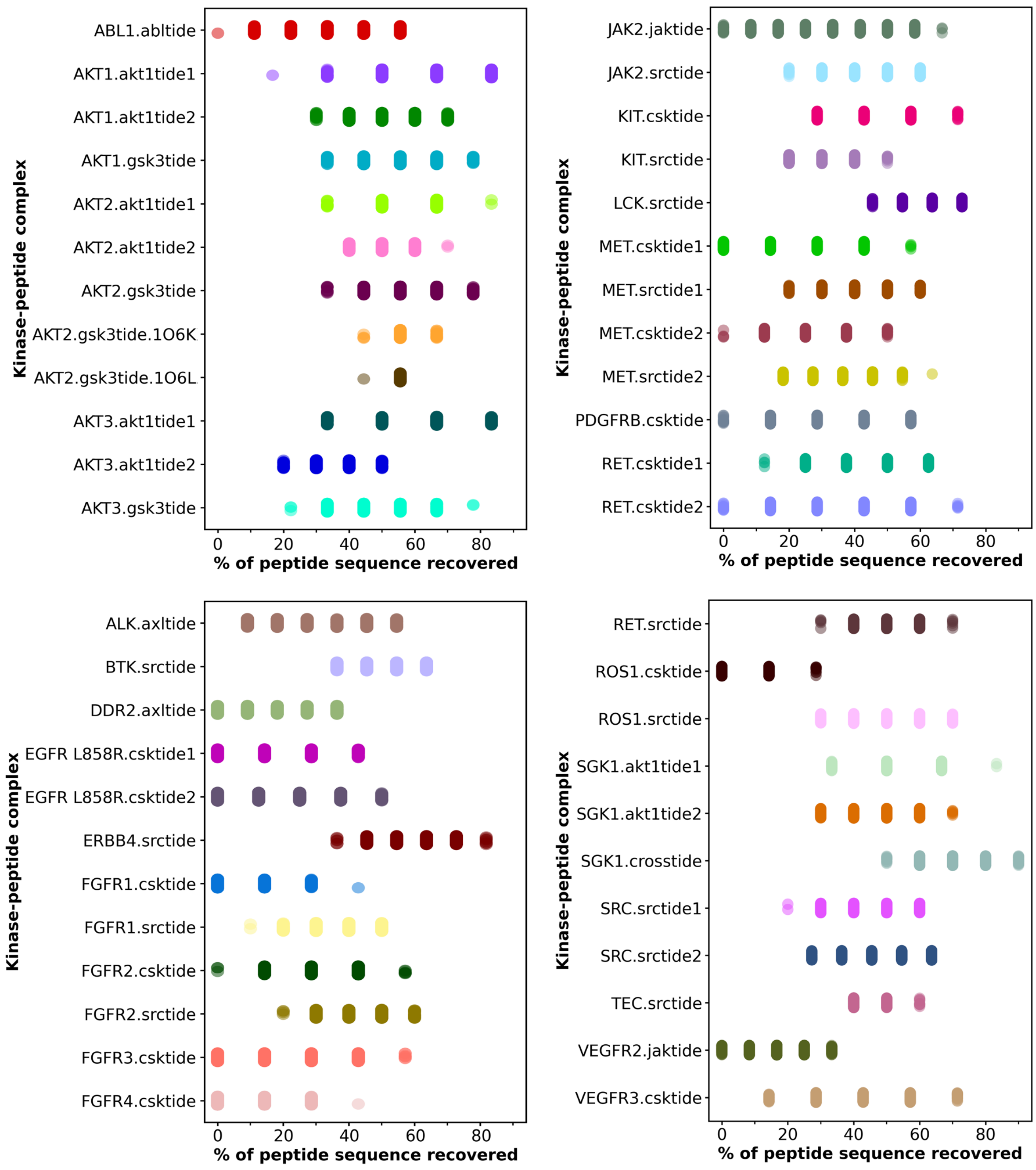

Figure S1: Substrate sequence recovery analysis across kinase families. Percentage of peptide sequence recovery for all 46 kinase-substrate pairs tested in the validation study. Each dot represents recovery percentage for individual Subtimizer designs. Most kinase-substrate pairs (>50%) achieve ≥70% sequence recovery, demonstrating robust identification of functionally important residues.

bioRxiv preprint doi: https://doi.org/10.1101/2025.07.04.663216; this version posted July 5, 2025. The copyright holder for this preprint (which was not certified by peer review) is the author/funder, who has granted bioRxiv a license to display the preprint in perpetuity. It is made available under aCC-BY 4.0 International license.

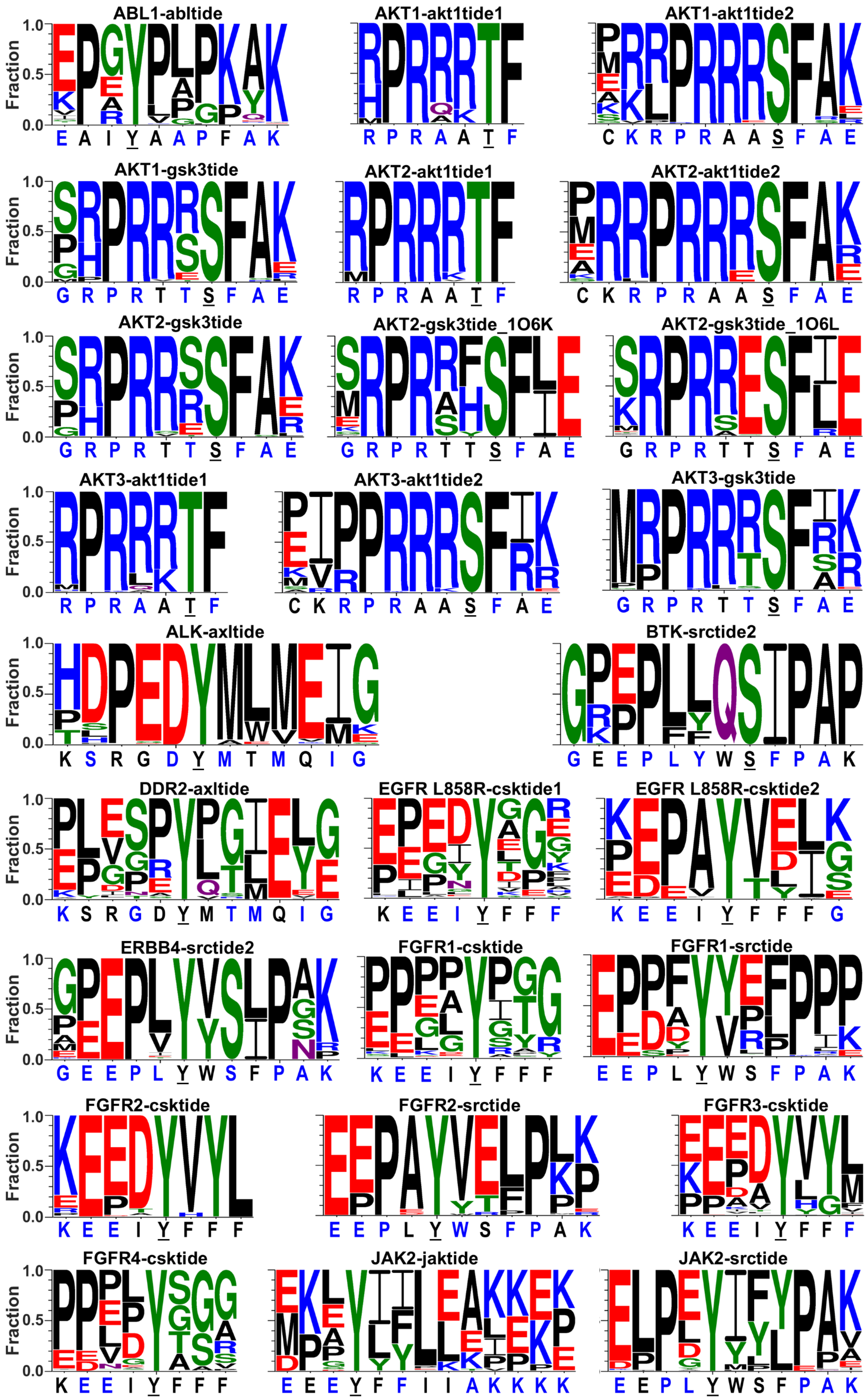

bioRxiv preprint doi: https://doi.org/10.1101/2025.07.04.663216; this version posted July 5, 2025. The copyright holder for this preprint (which was not certified by peer review) is the author/funder, who has granted bioRxiv a license to display the preprint in perpetuity. It is made available under aCC-BY 4.0 International license.

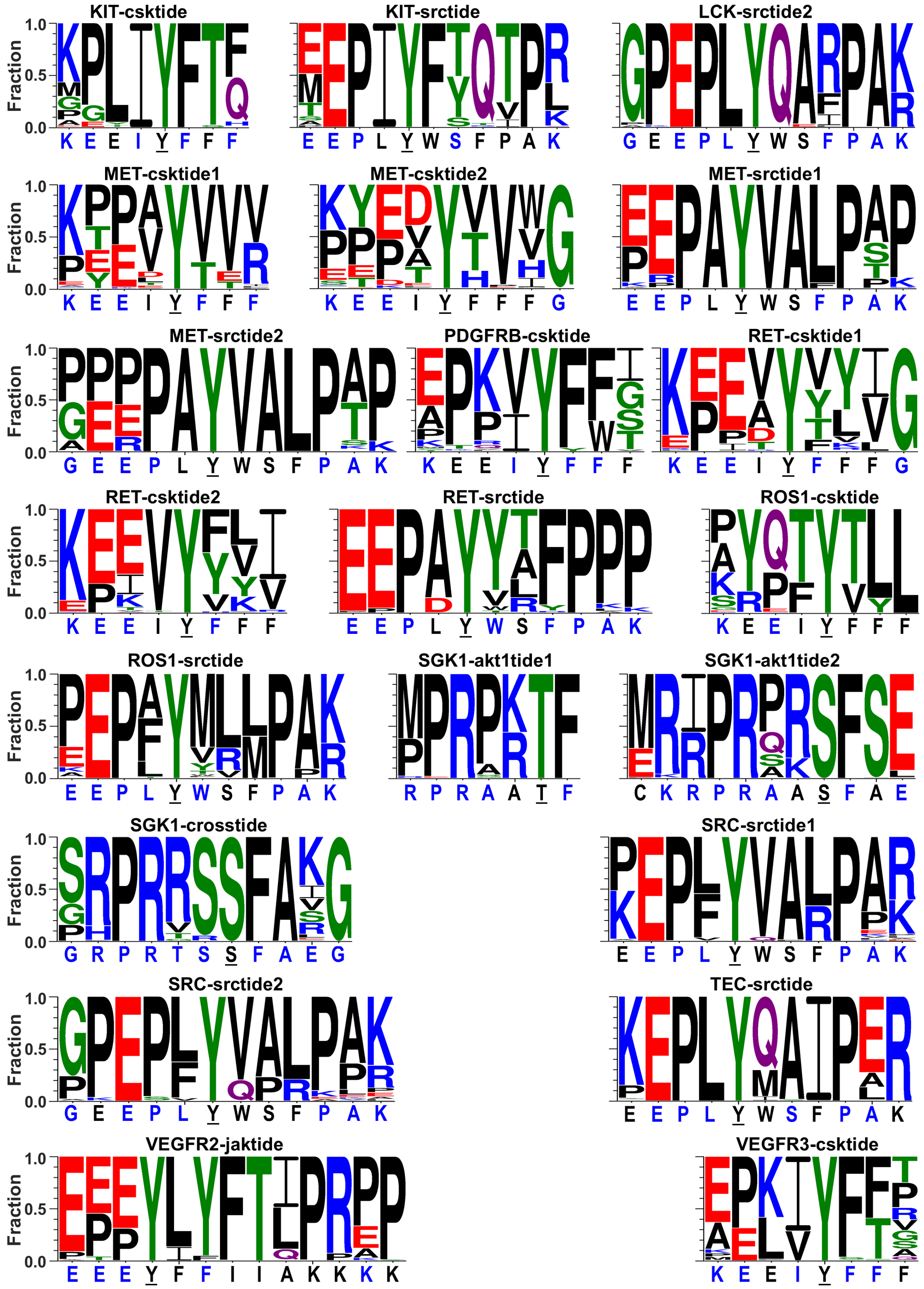

Figure S2: Sequence logos of Subtimizer-designed peptides. Amino acid frequency and preferences at each position for designed peptides across kinase-substrate pairs. Letter height corresponds to amino acid frequency. Phosphorylatable residues and structurally important positions show strong conservation, while variable positions indicate regions of sequence optimization.

bioRxiv preprint doi: https://doi.org/10.1101/2025.07.04.663216; this version posted July 5, 2025. The copyright holder for this preprint (which was not certified by peer review) is the author/funder, who has granted bioRxiv a license to display the preprint in perpetuity. It is made available under aCC-BY 4.0 International license.

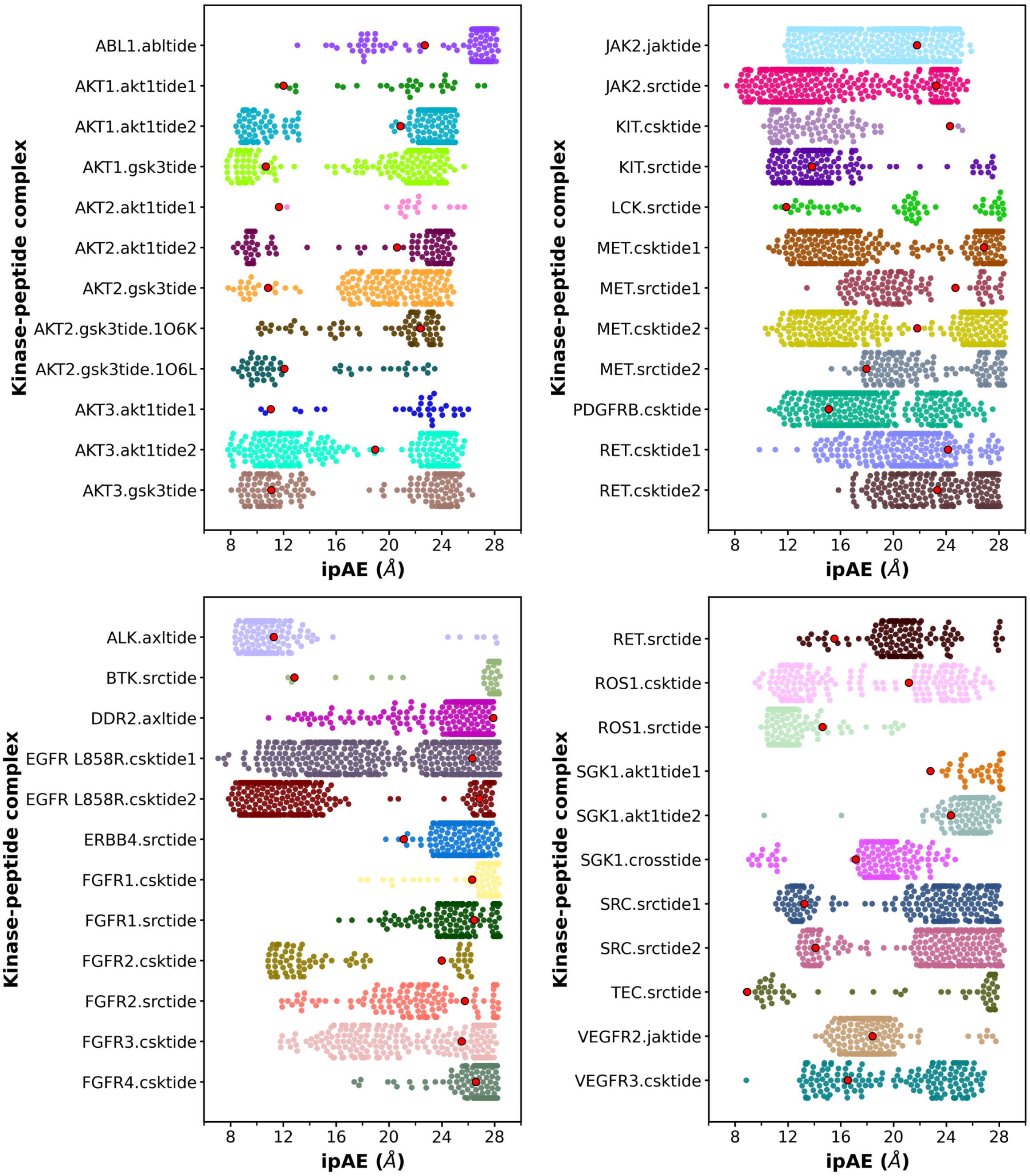

Figure S3: Distribution of interface predicted aligned error (ipAE) scores for all 47 kinase-substrate pairs. The ipAE scores of Subtimizer-designed peptides were compared with parental peptides (red dots). For 46 of the 47 pairs (98%), Subtimizer generated peptides with lower ipAE scores than parental substrates, with most achieving scores below 10 Å.

bioRxiv preprint doi: https://doi.org/10.1101/2025.07.04.663216; this version posted July 5, 2025. The copyright holder for this preprint (which was not certified by peer review) is the author/funder, who has granted bioRxiv a license to display the preprint in perpetuity. It is made available under aCC-BY 4.0 International license.

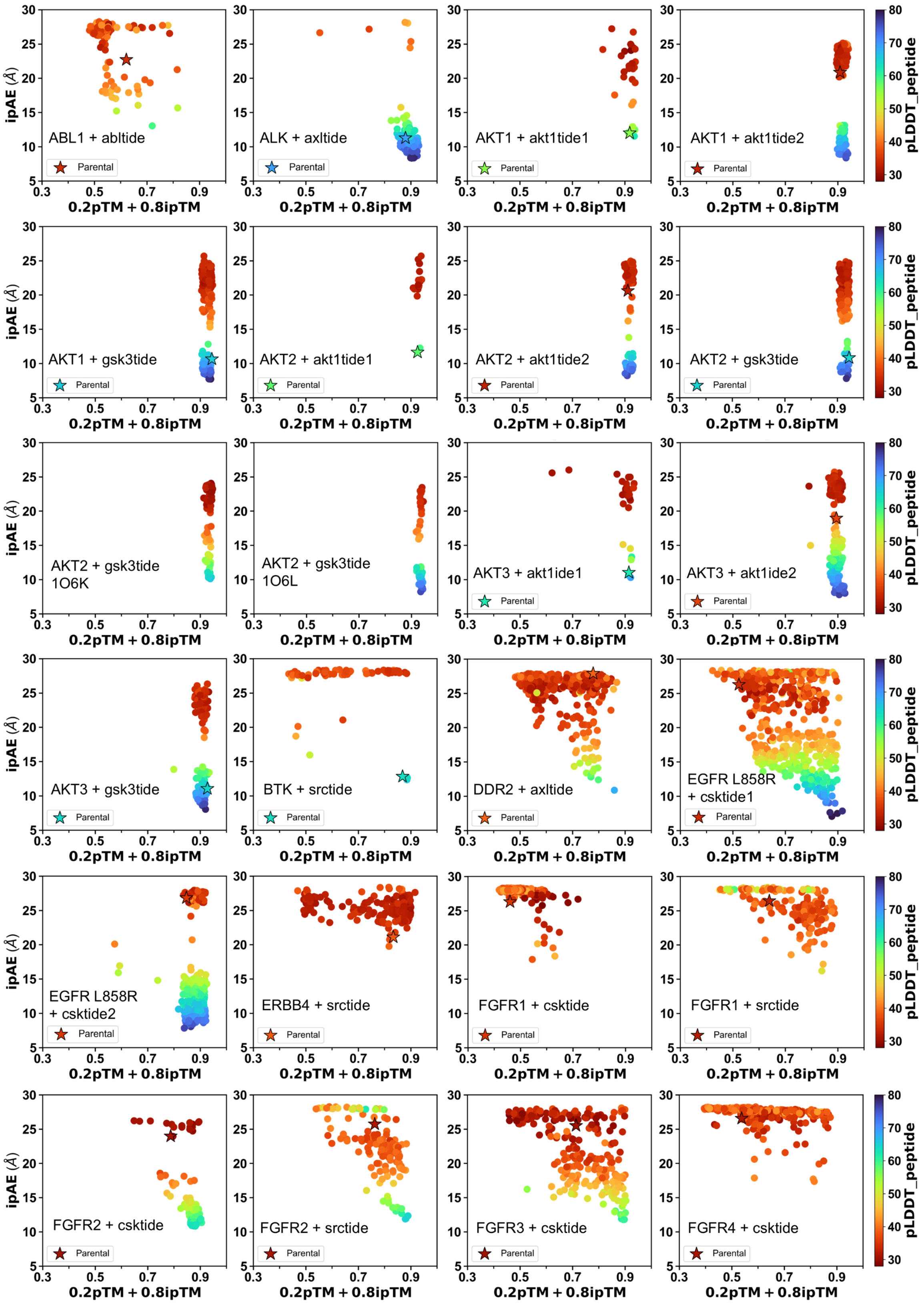

bioRxiv preprint doi: https://doi.org/10.1101/2025.07.04.663216; this version posted July 5, 2025. The copyright holder for this preprint (which was not certified by peer review) is the author/funder, who has granted bioRxiv a license to display the preprint in perpetuity. It is made available under aCC-BY 4.0 International license.

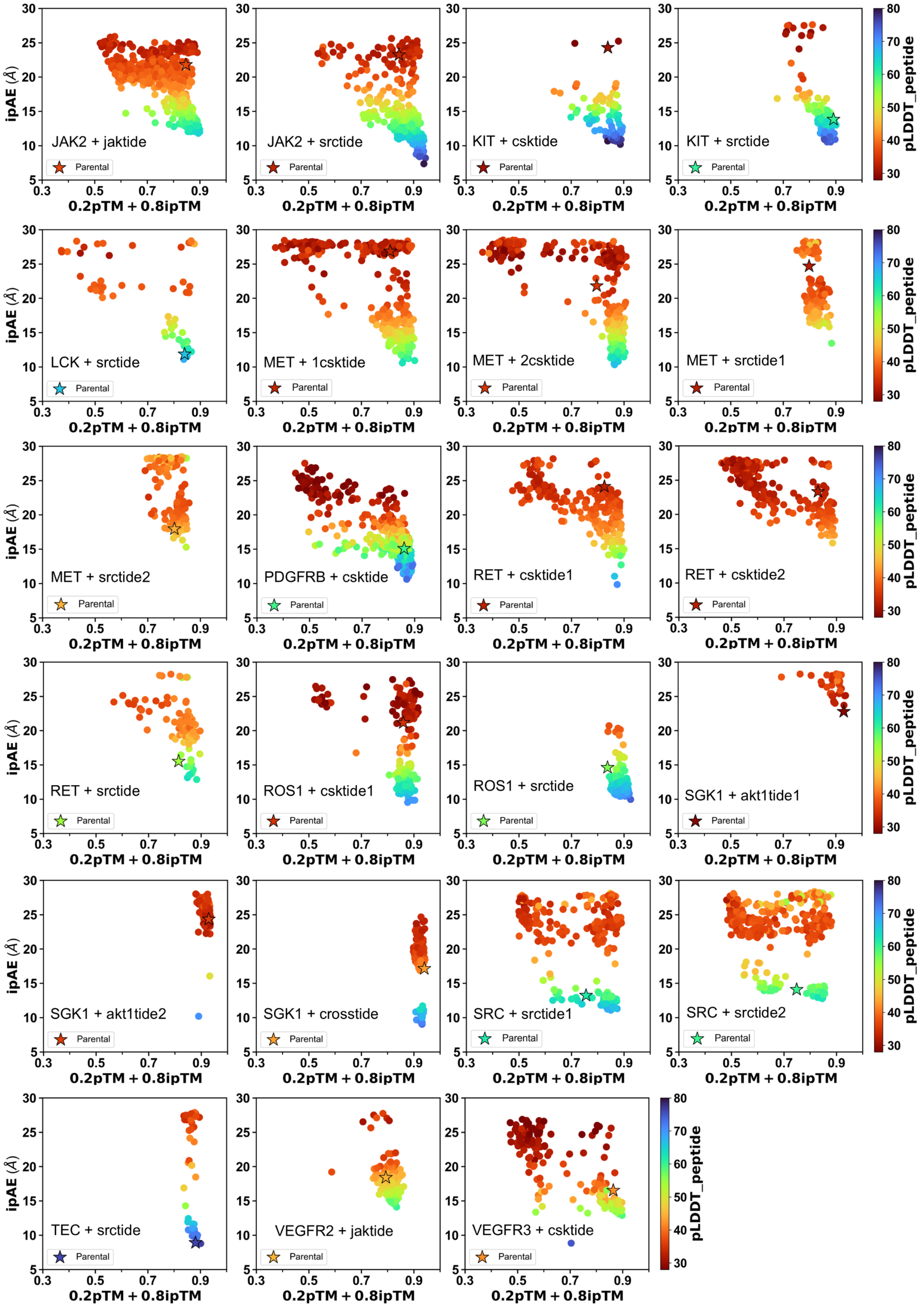

Figure S4: Correlation between structural evaluation metrics. Scatter plots showing relationship between ipAE and combined confidence scores ($0.2 \cdot \text{pTM} + 0.8 \cdot \text{ipTM}$) for all kinase-substrate pairs. Color gradient of dots show peptide confidence (pLDDT). Strong inverse correlation validates the use of these metrics for design evaluation.

bioRxiv preprint doi: https://doi.org/10.1101/2025.07.04.663216; this version posted July 5, 2025. The copyright holder for this preprint (which was not certified by peer review) is the author/funder, who has granted bioRxiv a license to display the preprint in perpetuity. It is made available under aCC-BY 4.0 International license.

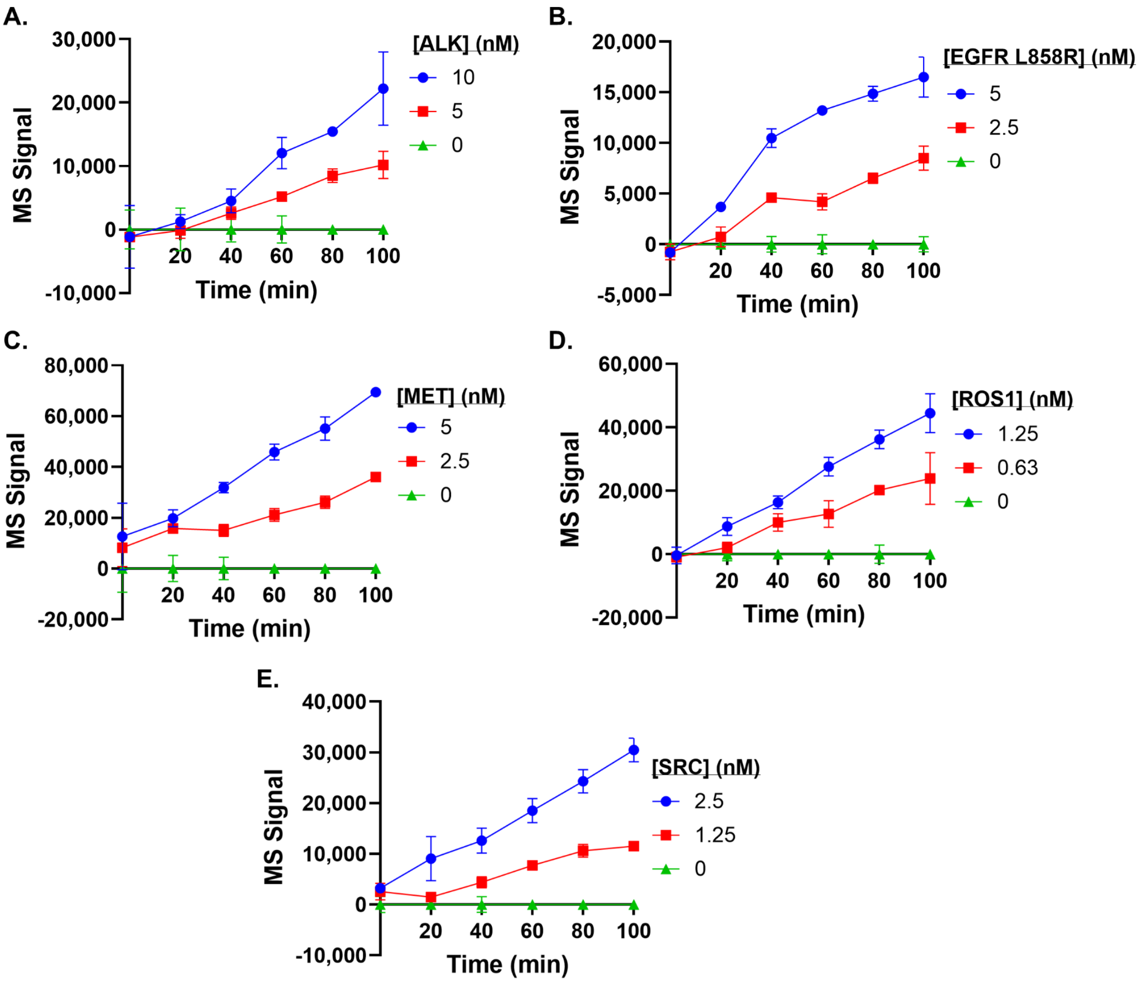

Figure S5: LIMS-kinase assay optimization confirms enzyme concentration- and time-dependent ADP production. Time-course analysis of ADP production across different enzyme concentrations for all five kinases tested, validating functional assay conditions for kinetic analysis. All assays performed with 100 μM ATP at varying peptide concentrations. Green triangles represent background controls (0 nM enzyme) showing minimal non-enzymatic ADP production. (**A**) ALK with 10 μM peptide substrate. (**B**) EGFR L858R with 1 μM substrate. (**C**) MET with 5 μM substrate. (**D**) ROS1 with 10 μM substrate. (**E**) SRC with 5 μM substrate.

bioRxiv preprint doi: https://doi.org/10.1101/2025.07.04.663216; this version posted July 5, 2025. The copyright holder for this preprint (which was not certified by peer review) is the author/funder, who has granted bioRxiv a license to display the preprint in perpetuity. It is made available under aCC-BY 4.0 International license.

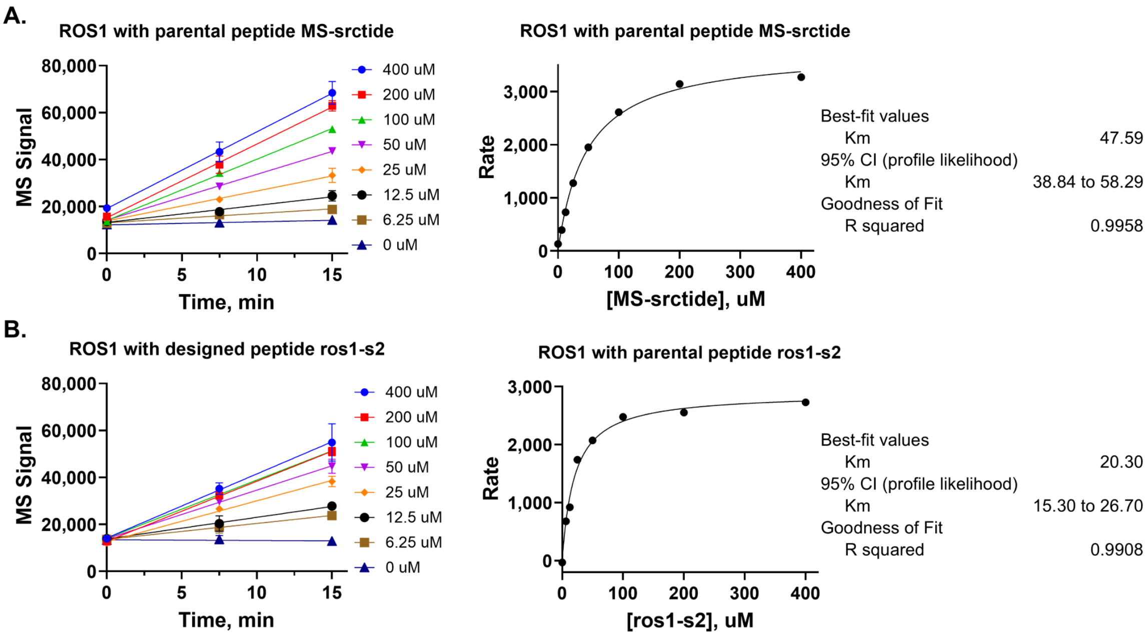

Figure S6: Detailed kinetic analysis of ROS1. (**A**) ROS1 kinetic analysis with parental peptide MS-srctide showing time-course measurements and Michaelis-Menten analysis (K_m = 47.59 μM, $R^2 = 0.9958$). (**B**) ROS1 analysis with designed peptide ros1-s2 (K_m = 20.30 μM, $R^2 = 0.9908$), demonstrating >2-fold improvement in apparent binding affinity.

bioRxiv preprint doi: https://doi.org/10.1101/2025.07.04.663216; this version posted July 5, 2025. The copyright holder for this preprint (which was not certified by peer review) is the author/funder, who has granted bioRxiv a license to display the preprint in perpetuity. It is made available under aCC-BY 4.0 International license.

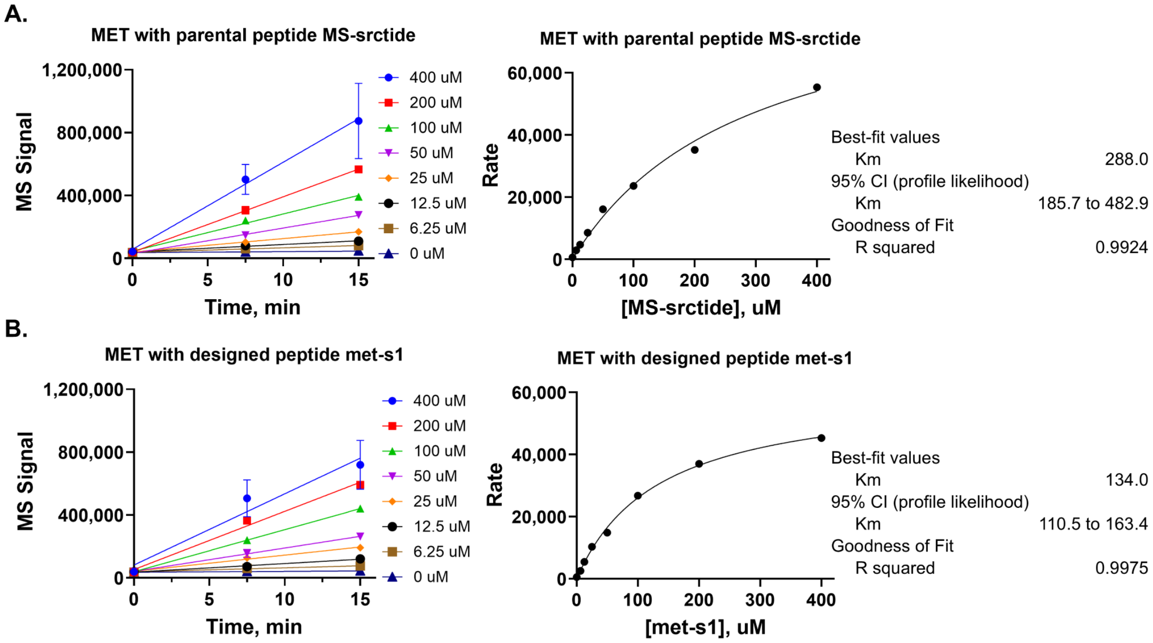

Figure S7: Kinetic analysis of MET kinase. (**A**) MET kinetic analysis with parental peptide MS-srctide showing concentration-dependent kinetics. (**B**) MET analysis with designed peptide met-s1, revealing improved saturation kinetics with >2-fold reduction in K_m (288 μM to 134 μM).

bioRxiv preprint doi: https://doi.org/10.1101/2025.07.04.663216; this version posted July 5, 2025. The copyright holder for this preprint (which was not certified by peer review) is the author/funder, who has granted bioRxiv a license to display the preprint in perpetuity. It is made available under aCC-BY 4.0 International license.

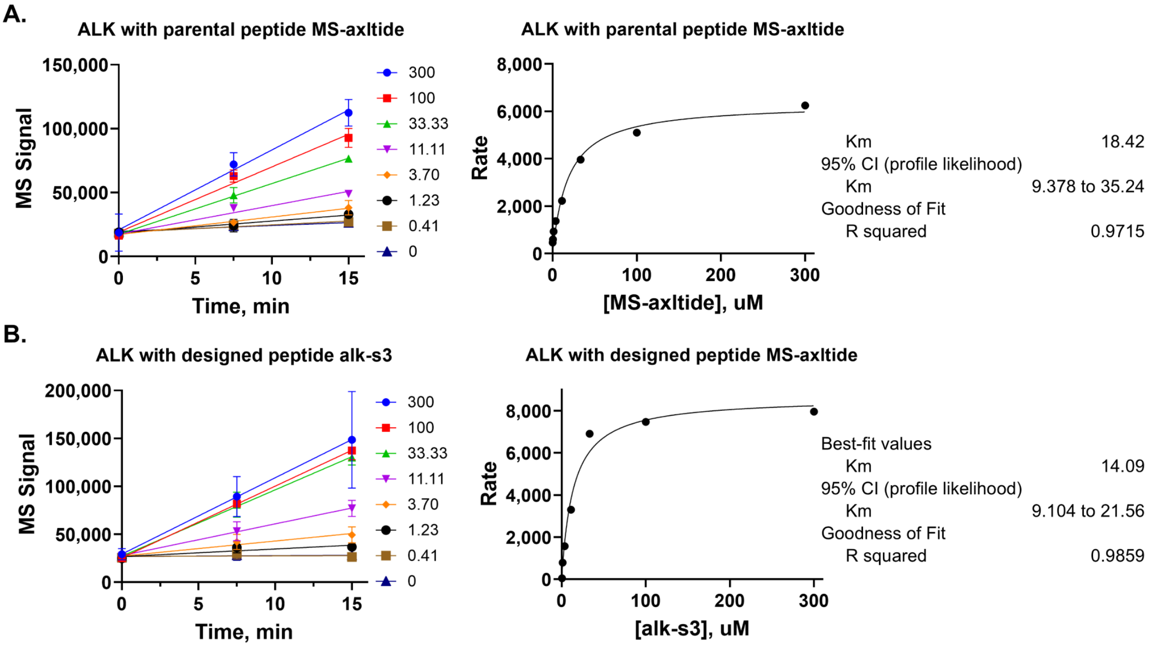

Figure S8: Kinetic characterization of ALK kinase. (**A**) ALK kinetic analysis with parental peptide MS-axltide. (**B**) ALK analysis with designed peptide alk-s3 showing improved binding affinity (K_m: 18.4 μM to 14.1 μM) and enhanced catalytic efficiency.

bioRxiv preprint doi: https://doi.org/10.1101/2025.07.04.663216; this version posted July 5, 2025. The copyright holder for this preprint (which was not certified by peer review) is the author/funder, who has granted bioRxiv a license to display the preprint in perpetuity. It is made available under aCC-BY 4.0 International license.

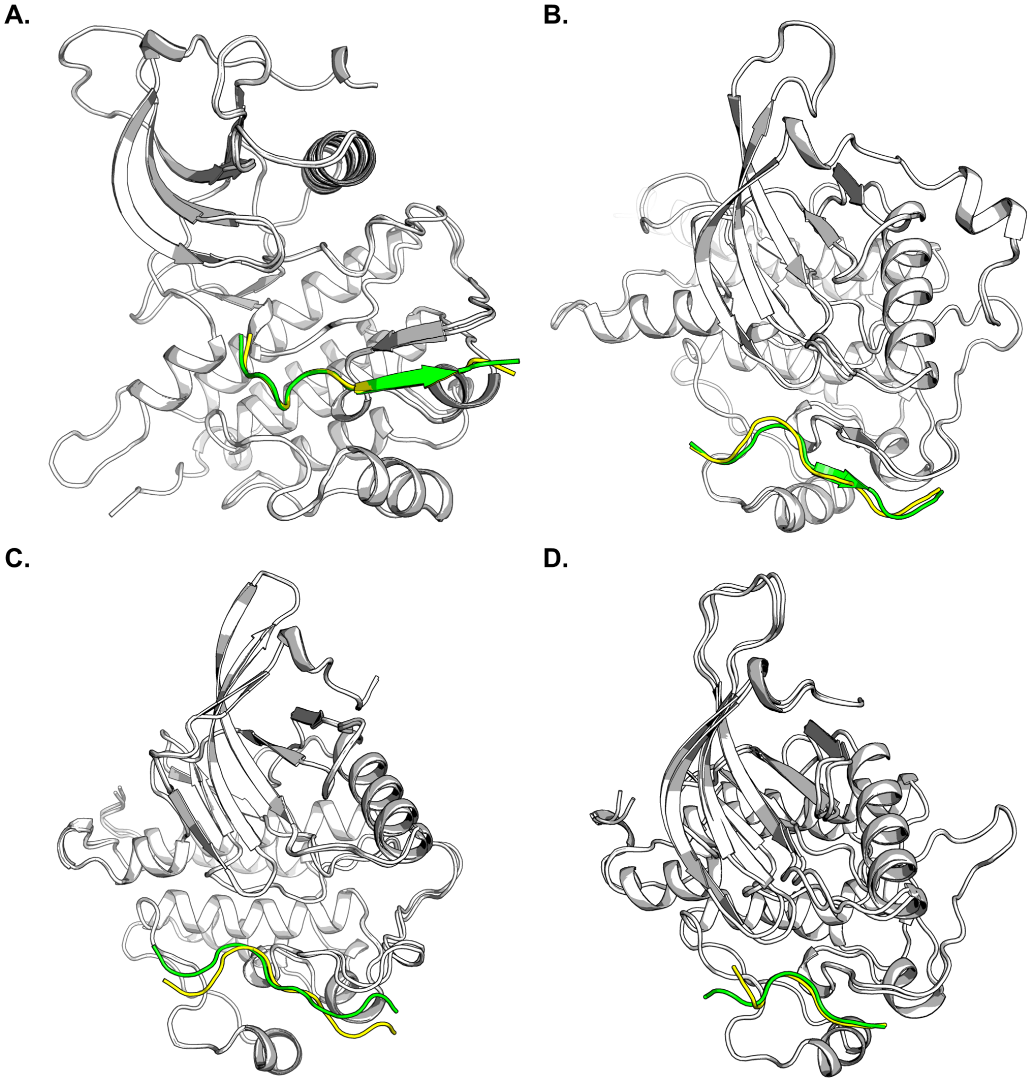

Figure S9: Structural comparison of AlphaFold-Multimer predicted structures of kinase-peptide complexes showing parental substrates (green) and Subtimizer-designed peptides (yellow) bound to the kinases (gray) ALK (**A**), ROS1 (**B**), MET (**C**), and EGFR L858R (**D**). Overlays reveal conformational differences and optimized binding modes in designed peptides.
